# Differences in the pharmacokinetics and steady-state blood concentrations of orally administered lenvatinib in adult and juvenile rats

**DOI:** 10.3389/fphar.2023.1140849

**Published:** 2023-07-28

**Authors:** Xiaoyue Du, Hongxin Cai, Nan Jin, Zhiguo Wu, Lele Wang, Zeyu Wang, Baogang Xie

**Affiliations:** ^1^ Jiaxing University Master Degree Cultivation Base, School of Pharmaceutical Sciences, Zhejiang Chinese Medical University, Hangzhou, China; ^2^ Medical College of Jiaxing University, Key Laboratory of Medical Electronics and Digital Health of Zhejiang Province, Jiaxing University, Jiaxing, China

**Keywords:** lenvatinib, pharmacokinetics, steady-state serum concentration, adult and juvenile rats, CYP3A2, SLC22A1

## Abstract

**Objective:** The aim of this study was to compare the pharmacokinetics and steady-state serum concentrations of lenvatinib in adult and juvenile rats.

**Experimental study:** An ultra-performance liquid chromatography-mass spectrometry (UPLC-MS) method was developed to quantify lenvatinib in the serum and liver of rats. Six juvenile and six adult rats in each group were orally administered with a single dose of 7.0 mg/kg lenvatinib suspension for pharmacokinetics. Another 12 juvenile and adult rats were subjected to oral gavage with 7.0 mg/kg lenvatinib once daily for 5 days. Biofluild samples were pre-treated by protein precipitation and sorafenib was used as the internal standard for UPLC-MS analysis. The pharmacokinetic parameters were estimated by compartment and statistical model. The mRNA expression of CYP3A2 and SLC22A1 in liver of adult and juvenile rats was measured by real-time fluorescence quantitative PCR (RT-qPCR).

**Results:** The UPLC-MS method met the requirements for quantitative analysis of lenvatinib in serum and liver. The pharmacokinetic results showed that the mean retention time (MRT_(0-∞)_) was 19.64 ± 7.64 h and 126.38 ± 130.18 h, with AUC_(0-∞)_ values of 3.97 ± 0.73 μg‧mL^-1^ h and 5.95 ± 2.27 μg mL^-1^ h in adult and juvenile rats, respectively. When comparing adult rats (0.35 ± 0.15 μg/mL) to juvenile rats, no significant differences were observed in steady-state serum lenvatinib (0.32 ± 0.11 μg/mL), but a noteworthy decrease to one-third of steady-state liver lenvatinib was observed after multiple oral doses of lenvatinib in juvenile rats. Additional findings revealed that the mRNA expression of CYP3A2 and SLC22A1 was notably increased by 6.86 and 14.67 times, respectively, in juvenile rats compared to adult rats.

**Conclusion:** Juvenile rats exhibit lower levels of lenvatinib in the liver’s steady-state, potentially due to the disparity in CYP3A2 mRNA expression. These results imply that the dosage of lenvatinib for pediatric patients may need to be augmented in order to attain the desired clinical outcome.

## 1 Introduction

Primary liver cancer including hepatocellular carcinoma (HCC) and intrahepatic cholangiocarcinoma and mixed hepatocellular carcinoma-cholangiocarcinoma, which ranks fifth in cancer incidence and second in mortality among all cancers in China (Chen et al., 2016; Siegel et al., 2020). According to the latest global cancer data released by the World Health Organization International Agency for Research on Cancer (IARC), 45.3% of new cases globally occurred in China in 2020 (Shi et al., 2021). Additionally, the incidence and mortality rates of liver cancer in China have shown a decreasing trend in the past few decades, which may be attributed to the effective control of hepatitis B virus (HBV) infection and aflatoxin food contamination (Torre et al., 2016; Benson et al., 2021). However, half of liver cancer patients succumb to tumor recurrence and metastasis in 5 years (Zeng et al., 2018). The situation of liver cancer prevention and treatment is still very urgent. Most of liver cancer patients in China cannot undergo surgery, ablation, transarterial chemoembolization (*TACE*) due to most of them being diagnosed at an advanced stage, systemic chemotherapy is the common palliative treatment in the clinic. Some cytotoxic drugs, including adriamycin, 5-fluorouracil and cisplatin, are the common chemotherapeutic agents employed for HCC treatment, whereas, no survival benefits and severe adverse effects of them, would be obtained, either alone or in combination. Treatment of HCC remains a major challenge in health cares.

The approval of sorafenib in 2007, a tyrosine kinase inhibitor, significantly prolonged the survival of patients with unresectable liver cancer (Chaparro et al., 2008), however, the drug resistance was found to be significant in subsequent treatments (Tang et al., 2020). Lenvatinib is effective against a wide range of cancers (Yamada et al., 2011; Boss et al., 2012) and was initially approved in Japan, the EU and the US for the treatment of advanced renal cell carcinoma and unresectable thyroid cancer (Schlumberger et al., 2015). In 2017, clinical trial studies showed that lenvatinib significantly prolonged median survival of advanced liver cancer patients compared with sorafenib. It was officially approved by the Food and Drug Administration (*FDA*) as a first-line target for unresectable liver cancer in 2018. Lenvatinib possesses more accurate targeting, stronger inhibition, lower side effects (Liu et al., 2019). Lenvatinib is a multi-targeted receptor tyrosine kinase (RTK) inhibitor, it inhibits vascular endothelial growth factor receptor 1 (VEGFR1), VEGFR2 and VEGFR3, as well as other RTKs associated with pathological neovascularization, tumor growth and cancer progression (Decraecker et al., 2021). Platelet-derived growth factor receptor alpha (PDGFRα), stem cell *factor receptor* (*KIT*) *and* rearranged during transfection (*RET*), are more effective for the treatment of patients with unresectable HCC (Faivre et al., 2020). HCC caused by hepatitis B (HBV) infection accounts for more than 90% of all cases in China. Lenvatinib is significantly more effective than sorafenib in HBV-related HCC and is more suitable for Chinese HCC patients, with a median survival of 13.6 months after treatment (Lu et al., 2019).

Cancer is one of the leading causes of death in children and adolescents globally, and the burden of cancer in children and adolescents in China is higher than in most of countries (Ni et al., 2022). To date, few reports on HCC treatment with lenvatinib was obtained in pediatrics. Differences in the biopharmacological properties of the drug in children and adults may lead to differences in their steady-state blood concentrations, which in turn might affect efficacy and toxicities. Here, a rapid, sensitive and accurate analytical method for the determination of lenvatinib in rat serum and liver by UPLC-MS was developed, differences in the pharmacokinetics of a single oral dose of lenvatinib and differences in steady-state serum concentrations after multiple doses were investigated in juvenile and adult rats. The results of this study contribute to the rational clinical use of lenvatinib in children.

## 2 Materials and experimental study

### 2.1 Experimental reagents

Lenvatinib (Shanghai Maclean Biochemical Co., Ltd.); sorafenib (Guangzhou Dongbang Pharmaceutical Technology Co., Ltd.); Acetonitrile and methanol (LC-MS grade, Beijing J&K Scientific Technology Co., Ltd.). PrimeScript™ RT Master Mix (Perfect Real Time), TB Green^®^ Premix Ex Taq™ (Tli RNaseH Plus) (Takara Bio, Japan). Isoflurane (Reward Life Sciences, China).

### 2.2 Animals

Adult (250–300 g) and juvenile (80–100 g) Sprague-Dawley (SD) rats, were purchased from Zhejiang Vital River Laboratory Animal Technology Co., Ltd., production license No. SCXK (Zhe) 2019-0001. The animals were raised in a specific pathogen-free environment. All animal experimental protocols were conducted after passing the review of the Animal Ethics Committee of Jiaxing University.

### 2.3 Sample preparation for LC-MS analysis

Orbital venous blood was collected and allowed to stand for 2 h at room temperature. The serum was obtained by centrifuging at 3,000 r for 5 min and kept in a refrigerator at −80°C for further analysis. 200 μL of methanol was added to 50.0 μL of serum in a 1.5 mL centrifuge tube and mixed by vortex. The mixture was then centrifuged at 13,000 r for 5 min, subsequently, 200 μL of supernatant was blent with 20 μL of sorafenib standard solution (2 μg/mL) as internal standard (IS) for sampling.

The 0.20 g liver samples were homogenized in 4.0 mL 80% methanol and centrifuged at 13,000 r for 10 min to collect the supernatants. 20.0 μL of IS was added to 200 μL of the supernatant for further UPLC-MS analysis.

### 2.4 UPLC-MS conditions and parameters for measuring lenvatinib

An UltiMate 3,000 ultra-high-performance liquid chromatography system consisting of an LPG-3400SD pump and a single-quadrupole mass spectrometer (Thermo *Fisher* Scientific) was used in this study. The samples were separated by an ACOQUITY UPLC^®^ BEH C18 column (1.7 μm, 2.1 × 50 mm), pre-column (Hypersil GOLD C18, 10 × 4.0 mm 3 μm) (Thermo Fisher Scientific, United States). The chromatographic gradient elution conditions as follows: 0.01% formic acid water (phase A) - acetonitrile (phase B); 0–0.5 min, 90% A; 0.5–1.5 min, 90%–5% A; 1.5–7.0 min, 5% A; 7.0–8.0 min, 5%–90% A; 8.0–13.0 min, 90% A; flow rate was set 0.30 mL/min and the column temperature is 45°C.

MS detection was achieved by the electrospray ion (ESI) source in the positive model; IS, lenvatinib, its metabolites M2 and M3 (Vavrova et al., 2022) were detected using selected ion monitoring (SIM) model the [M + H]^+^, m/z 427.1, 413.1 and 443.1 respectively. The MS condition is as follows: spray voltage (Source voltage positive, 3,000 V; sheath gas pressure, 35.8 psi; auxiliary gas pressure, 4.0 psi; Ion transfer tube temperature, 300°C; Vaporizer temperature, 172°C.

### 2.5 UPLC-MS method validation

Blank serum, lenvatinib and IS solution, and serum of rats after lenvatinib administration were analyzed to determine the specificity of the method. Eight standard solutions of lenvatinib were prepared from the stock solutions in 80% methanol to generate calibration curves from 1.18 ng/mL ∼ 1.14 μg/mL. The calibration curve was obtained by ratios between the lenvatinib peak area and the IS peak area against the corresponding lenvatinib concentrations. The limit of detection (LOD) is defined as the lowest lenvatinib concentration resulting in a signal-to-noise ratio of 3:1, whereas the limit of quantification (LOQ) is the sample resulting in a signal-to-noise ratio of 10:1. To obtain the intra-day, inter-day precision and long-time stability, a set of serum and liver samples containing lenvatinib were selected and placed at room temperature in 12, 24, and 48 h as well as −20°C for 15 days, respectively.

The method recovery was based on the recovery of known concentrations of analyse by spiking the standard at high, medium, and low concentration into the serum and liver. Matrix effects were determined by comparing *the* peak areas of standard lenvatinib solution between spiked in serum and spiked in the mobile phase.

### 2.6 Pharmacokinetic study of lenvatinib after oral administration in adult and juvenile rats

Six juvenile male Sprague-Dawley (*SD*) rats (4 weeks) and six adult SD rats (8 weeks) were orally gavaged with 2.0 mL lenvatinib suspension in 0.15% (w/v) carboxymethyl cellulose (*CMC*) solution (7.0 mg/kg). After treatment, 0.5 mL of blood was taken from the fundus of the eye at 0.5, 1.0, 2.0, 4.0, 6.0, 8.0, 10.0, 12.0, 24.0, 36.0, 48.0, and 72.0 h. The serum was obtained and stored at −80°C for further analysis.

### 2.7 Steady-state serum and liver drug concentrations in adult and juvenile rats after multiple oral doses of lenvatinib

Six juvenile male SD rats (4 weeks) and six adult SD rats (8 weeks) were orally gavaged with 2.0 mL lenvatinib suspension in 0.15% (w/v) CMC solution (7.0 mg/kg) once daily for five consecutive days. At 2 h after the completion of gavage on day five, 2.0–3.0 mL of blood was taken from the fundus of each rat and placed in a centrifuge tube at room temperature for 2 h. After centrifugation at 3,000 rpm for 5 min, the serum was collected and stored at −80°C. Then rats were sacrificed by cervical dislocation, liver tissue was removed and stored at −80°C.

### 2.8 Liver expression of CYP3A2 and SLC22A1 mRNA in adult and juvenile rats

Frozen rat liver tissues were ground in liquid nitrogen and the RNA was extracted using Trizol reagent. The purity, integrity, and concentration of RNA were quantified using a NanoDrop 2000 spectrophotometer (Thermo Fisher Scientific, United States). Reverse transcription of RNA to cDNA process was performed by MiniAmp™ Thermal Cycler (Thermo Fisher Scientific, United States). All PCR reactions were performed in 10 μL reaction volumes with 2 μL of RNA added per reaction. Real Time PCR with TB Green™ Premix Ex Taq™ was performed according to Takara’s TB Green chimeric fluorescence method. The master mix contained: 0.5 μL forward primer (10 mM), 0.5 mL reverse primer (10 mM), 5.0 μL TB Green Premix Ex Taq (Tli RNaseH Plus) (TaKaRa BIO, Shiga, Japan), 2.0 μL deionized distilled water (dd H2O) and 2.0 μL cDNA (15.0 ng/μL). The PCR mix was subjected to PCR in a Mastercycler ep realplex (Eppendorf, Hamburg, Germany). An initial denaturation was performed at 95°C for 2 min, followed by 45 cycles of 95°C for 30 s, the annealing 60°C for 30 s, and a final extension 68°C for 30 s. The relative expression of the target genes was calculated using the 2^−ΔΔCt^ method. The primer sequences of GAPDH, CYP3A2, and SLC22A1 were designed by Boshang Biotechnology (Shanghai) Co., Ltd.) and were shown in Table 1.

**TABLE 1:** Primer sequences for determination of gene expression levels.

Gene	Forward primer (FP)	Reverse primer (FP)	Melting (°C)	Product size
GAPDH	GCC​ATC​ACT​GCC​ACT​CAG​AAG​A	ATA​CAT​TGG​GGG​TAG​GAA​CAC​G	60	184
CYP3A2	CCA​CGT​TCA​CCA​GTG​GAA​GA	CTC​CGC​CTC​TTG​CTT​CAA​GT	60	182
SLC22A1	GTTACCCTCGCCTGTCTT	CTTGCCAAACTTCCATCA	53.4	184

### 2.9 Statistical analysis

The pharmacokinetic parameters of lenvatinib after oral administration in rats were estimated by DAS 2.0 pharmacokinetic software (Chinese Pharmacological Association, China). Statistical analysis was performed using IBM SPSS for Windows (Statistical Package for the Social Sciences), version 26.0 software package for Macintosh (SPSS, Inc., Chicago, IL, United States). One-way ANOVA and Tukey’s multiple comparison tests were used to analyze the natural logarithm (ln)-transformed pharmacokinetic parameters (AUC_0–t_, AUC_0–∞_, and C_max_) by gen linear model procedures. Quantitative data were expressed as mean ± variance (mean ± SD), and independent samples t-tests or Mann-Whitney test were used for comparison between two groups by Graphpad Prism 8.0 (Graphpad Software, San Diego, CA). A value of *p* < 0.05 was considered statistically significant.

## 3 Results

### 3.1 Development of LC-MS method for measuring lenvatinib in serum and liver

**FIGURE 1:**
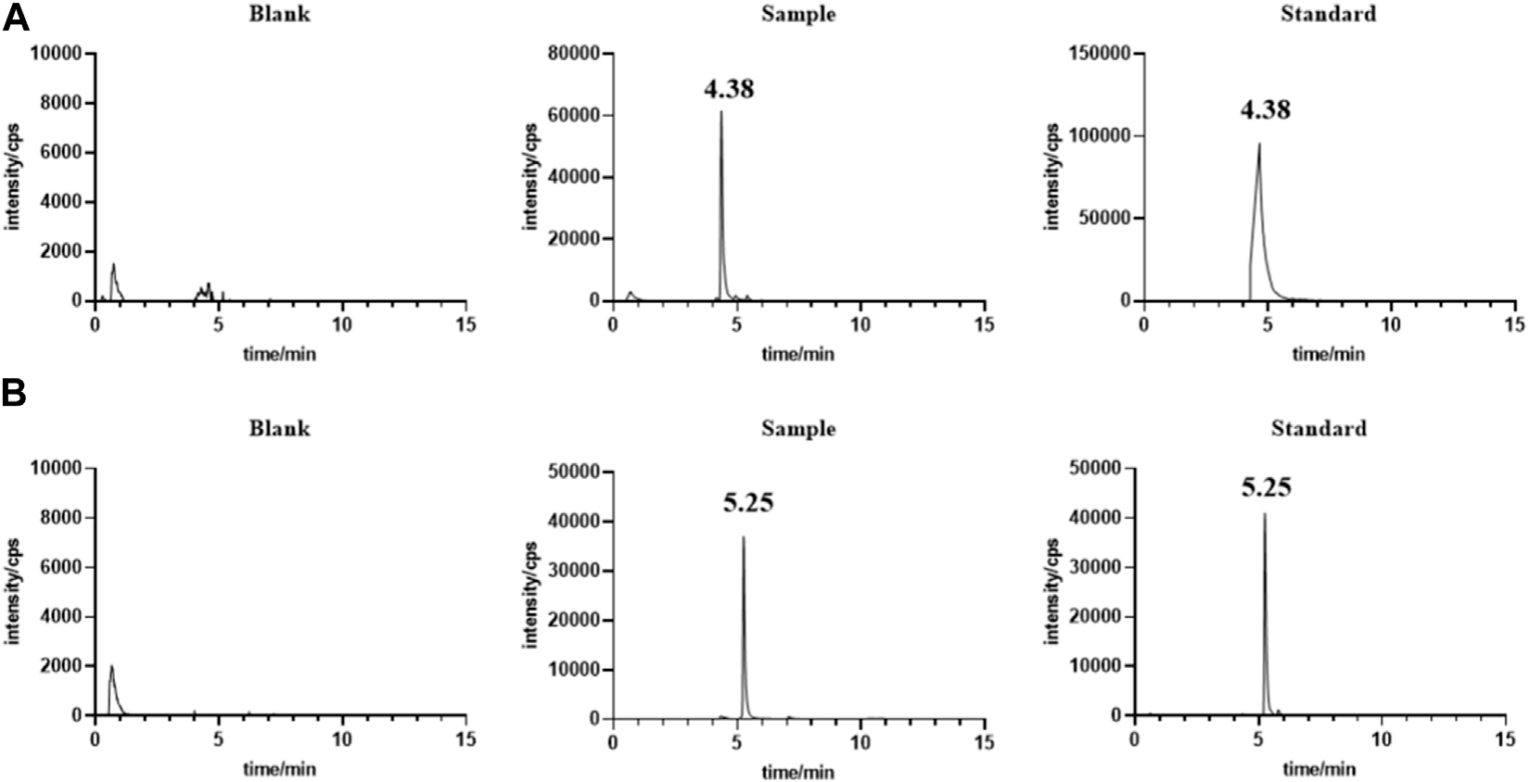
Representative chromatograms for lenvatinib **(A)** and IS **(B)** in rat serum samples.

Under the chromatographic conditions, endogenous substances in serum (Figure 1) and liver (Figure not shown) did not interfere with the determination of the target drug, indicating the high specificity of the method. *The* calibration curve from 1.18 ng/mL ∼ 1.14 μg/mL has a *good* linear response with the square of *correlation coefficient* (*R* ^2^) over *0.999*. The LOD and LOQ were 0.06 ng/mL and 1.8 ng/mL, respectively. The intra-day precision was less than 7.8% and the inter-day precision was less than 9.1% in serum and liver respectively. The mean method recoveries were 127.41%–138.82% and 130.62%–149.70%, respectively, and the matrix effects were 96.97%–100.68%, 103.31%–112.48% in serum and liver respectively, as shown in Table 2. Our results showed that the storage of the serum or extract of liver samples for 48 h at 4°C and −20°C for 15 days exerted little effect (RSD less than 15.0%). In summary, our results indicated the developed UPLC-MS method could meet the requirement of biological analysis.

**TABLE 2:** Matrix effect and extraction recovery of lenvatinib in rat serum and liver (n = 3).

Analytes	Concentration (μg/mL)	Matrix effect			Method recovery	
		Mean ± SD (%)	RSD (%)	Mean ± SD (%)		RSD (%)
	2.5	100.06 ± 0.35	0.35	138.82.06 ± 2.17		1.63
serum	1.25	100.68 ± 0.71	0.70	127.41 ± 1.88		1.47
	0.625	96.97 ± 1.42	1.46	138.26 ± 0.23		0.17
	2.5	103.31 ± 1.00	0.96	130.62 ± 2.82		2.16
liver	1.25	106.17 ± 1.80	1.70	139.41 ± 1.10		0.79
	0.625	112.48 ± 8.36	7.43	149.70 ± 1.32		0.88

### 3.2 Pharmacokinetic study of oral administration of lenvatinib in adult SD and juvenile rats

**FIGURE 2:**
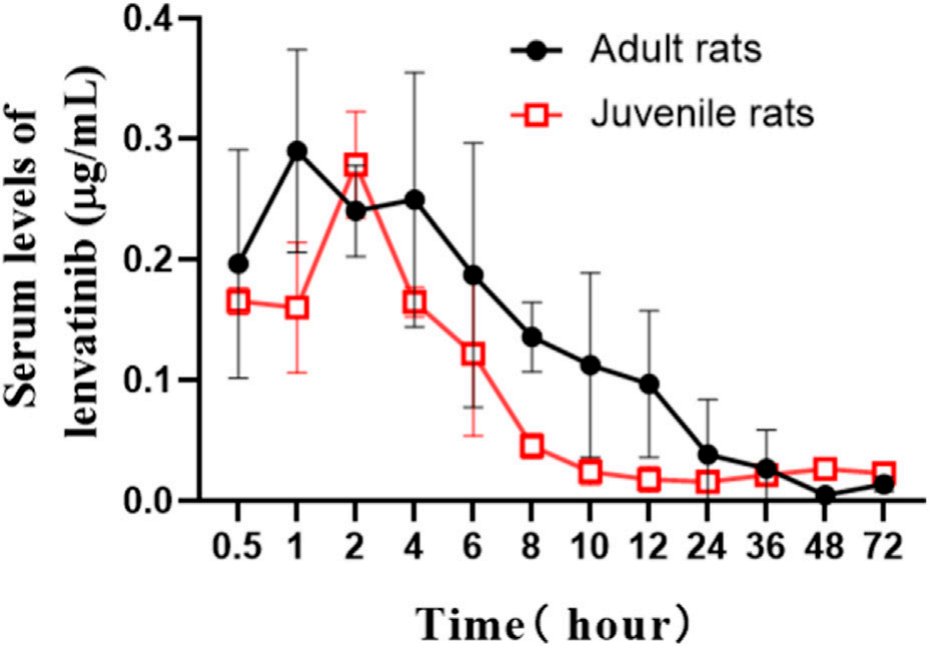
The mean serum concentration-time curves of lenvatinib after oral administration (7.0 mg/kg) in adult and juvenile rats.

The serum concentrations of lenvatinib over time after oral gavage administration of lenvatinib suspension (7.0 mg/kg) in SD adult and juvenile rats are shown in Figure 2. The results of the statistical moment model showed that in adult and juvenile rats, the mean retention time (MRT_(0-∞)_) was 19.64 ± 7.64 h and 126.38 ± 130.18 h, respectively. When compared with adult rats, about 6 times increase was observed of MRT_(0-∞)_ in juvenile rats. The values of CL/F were less than about 30% in juvenile rats than those in adult rats. In addition, the maximum serum concentration (C_max_) was 0.32 ± 0.08 μg/mL and 0.28 ± 0.04 μg/mL in adult rats and juvenile rats, respectively; the area under the drug-time curve (AUC_(0-∞)_) was 3.97 ± 0.73 μg/mL h and 5.95 ± 2.27 μg/mL h, respectively, indicating that there is no significant difference between adult and juvenile rats in the relative oral bioavailability of lenvatinib. The Pharmacokinetic parameters of lenvatinib in adult and juvenile rats are shown in Table 3.

**TABLE 3:** Pharmacokinetic parameters of lenvatinib in adult and juvenile rats (7.0 mg/kg, means ± SD., *n* = 5–6).

Analysis mode	Adult rat	Juvenile rat
Two compartment model		
T_1/2α_(h)	216.58 ± 310.32	^a^104.35 ± 224.20
T_1/2β_(h)	45,376.84 ± 52,704.20	25,495.5 ± 44,224.25
T_1/2Ka_(h)	191.35 ± 299.97	**2.45 ± 3.38
V_1_/F (L·kg^-1^)	10.14 ± 12.15	^a^18,549.10 ± 41,446.83
CL/F (L·h^-1^·kg^-1^)	2.25 ± 0.68	2.71 ± 1.95
AUC_(0-∞)_ (μg·mL^-1^·h)	3.35 ± 0.96	4.15 ± 3.10
*R* ^2^	0.83 ± 0.14	0.83 ± 0.10
Statistical moments		
T_1/2_(h)	13.80 ± 4.28	^a^80.79 ± 105.80
V/F (L·kg^-1^)	35.82 ± 12.18	^a^109.78 ± 95.17
CL/F (L·h^-1^·kg^-1^)	1.82 ± 0.34	^a^1.28 ± 0.35
AUC_(0-∞)_ (μg·mL^-1^·h)	3.97 ± 0.73	5.95 ± 2.27
AUMC_(0-∞)_	78.98 ± 37.29	^a^985.10 ± 1,445.02
MRT_(0-∞)_ (h)	19.64 ± 7.64	**126.38 ± 130.18
C_max_ (μg·mL^-1^)	0.32 ± 0.08	0.28 ± 0.04
T_max_(h)	3.5 ± 0.73	^a^2.0 ± 0.00

T_1/2_, half-life; V/F, apparent volume of distribution corrected for extravascular bioavailability; CL/F, clearance corrected for extravascular bioavailability; AUC_(0–∞),_ area under the 0–∞ drug-time curve; AUMC_(0-∞),_ area under the 0-∞ first-order moment curve; MRT _(0-∞)_, 0–∞ mean retention time; C_max,_ peak concentration; T_max_: time to peak concentration.

^a^ Indicates statistically significant difference in lenvatinib compared to juvenile rats using independent sample *t*-test i.e., *indicates *p* < 0.05,**indicates *p* < 0.01.

### 3.3 Significant differences of steady-state liver lenvatinib levels in adult and juvenile rats

**FIGURE 3:**
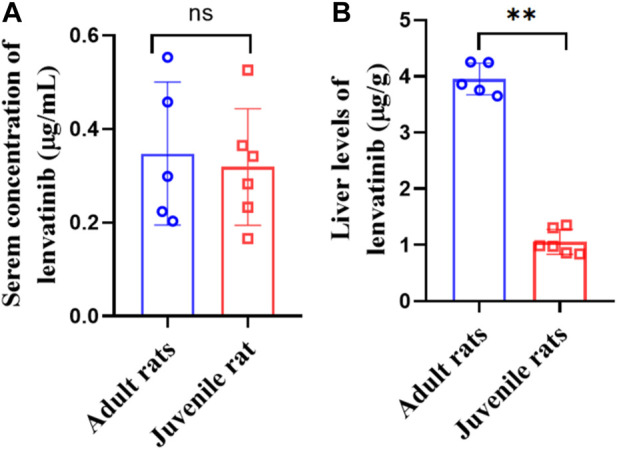
Serum **(A)** and liver **(B)** levels of lenvatinib in steady-state after multiple oral doses of lenvatinib (7.0 mg/kg).

After multiple oral administration of lenvatinib for five consecutive days, serum lenvatinib levels were 0.35 ± 0.15 μg/mL and 0.32 ± 0.11 μg/mL, and liver levels were 3.95 ± 0.28 μg/mL and 1.05 ± 0.20 μg/mL in adult and juvenile rats, respectively (Figures 3A, B). Significant decreases of steady-state drug concentrations in the liver were observed in juvenile rats compared with those observed in adult rats. The liver/serum content ratios were 13.2 ± 5.57 and 3.61 ± 1.05 mL/g in adult and juvenile rats, respectively (Table 4). Interestingly, significant increases in serum metabolite of lenvatinib were observed in juvenile rats compared with that observed in adult rats (Table 4), indicating the differences in lenvatinib metabolism between juvenile and adult rats.

**TABLE 4:** Steady-state liver and serum lenvatinib concentrations and two metabolites in adult and juvenile rats after multiple oral doses of lenvatinib (7.0 mg/kg, Mean ± S.D., n = 5–6).

	Adult rats			Juvenile rats		
	Liver (μg/g)	Serum (μg/mL)	Liver/serum	Liver (μg/g)	Serum (μg/mL)	Liver/serum
lenvatinib	3.95 ± 0.28	0.35 ± 0.15	13.2 ± 5.57	1.05 ± 0.20**	0.32 ± 0.11	3.61 ± 1.05^a^
M2 (peak area)	120.69 ± 4.60	99.43 ± 7.19	0.40 ± 0.049	25.56 ± 5.20**	130.92 ± 9.51**	0.20 ± 0.55**
M3 (peak area)	480.66 ± 68.99	490.78 ± 20.11	0.98 ± 3.43	384.09 ± 53.09**	537.39 ± 31.38**	0.71 ± 1.69

^a^ Indicates statistically significant difference of lenvatinib levels in juvenile rats compared to those in adult ones using independent sample *t*-test i.e.,**indicates *p* < 0.01.

### 3.4 Elevated CYP3A2 and SLC22A1 mRNA expression in the liver of juvenile rats

**FIGURE 4:**
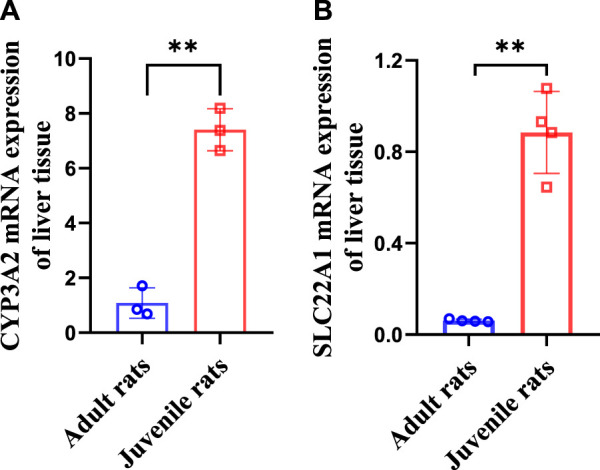
Differences in CYP3A2 **(A)** and SLC22A1 **(B)** mRNA levels of liver tissues between adult and juvenile rats.

Our results (Figure 4) showed that the expression of CYP3A2 in mRNA level was 1.08 ± 0.45 and 7.41 ± 0.63 in adult and juvenile rats respectively, which was significantly upregulated by 6.86 times in juvenile rats compared to adult rats. The expression of SLC22A1 in mRNA level was 0.06 ± 0.01 and 0.88 ± 0.16 in adult and juvenile rats respectively. SLC22A1 was significantly upregulated by 14.67 times in juvenile rats compared to adult rats.

## 4 Discussion

The LC-MS method is a simple, cost-effective, and reliable technique for the quantitative evaluation of lenvatinib in biological matrices (Srikanth and Prameela, 2017). Employed liquid-liquid extraction and LC-MS/MS method for the determination of plasma lenvatinib (I and A, 2017). Ogawa-Morita T et al. developed an LC-MS/MS method to detect lenvatinib in human serum with an API3200 quadrupole mass spectrometry (Ogawa-Morita et al., 2017). However, the instrument of LC-MS/MS was generally not available in some clinical laboratories. In this study, the UPLC-MS method was developed with an ACOQUITY UPLC^®^ BEH C18 column for the separation of lenvatinib in rat serum and liver samples. The samples were directly loaded after the precipitation of the proteins using methanol. The retention times of lenvatinib and IS were 4.38 and 5.25 min, respectively. The methodological investigation showed that the developed UPLC-MS method was satisfactory for the requirement of biological analysis, which can be considered a more economical and feasible method for measuring serum and liver lenvatinib levels. Cui et al. (2021) developed a rapid and sensitive LC-MS/MS method for the pharmacokinetic study of lenvatinib in rats, which yielded AUC_(0-∞)_ of 3.37 ± 0.98 mg/L h and C_max_ of 0.49 ± 0.12 mg/L for 1.2 mg/kg lenvatinib by oral gavage. However, no study focuses on the differences of pharmacokinetics and steady-state blood and liver levels of lenvatinib between adult and juvenile rats.

Considering that sexual maturation begins after 6 weeks in rats (Donner et al., 2015; Sengupta, 2013), here we chose 4-week-old and 8-week-old rats to investigate the differences of pharmacokinetics and steady-state blood concentrations in age after oral lenvatinib treatment. Our results suggest that the pharmacokinetics of lenvatinib in rats conformed to a two-compartment open model. A three-compartment model with linear elimination was observed in humans after oral administration of (Gupta et al., 2016). This discrepancy *may be due to* the species *differences* in pharmacokinetic characteristics between rats and *humans. Our results showed that the* AUC_(0-∞)_ values of lenvatinib were 3.35 ± 0.96 μg/mL h and 3.97 ± 0.73 μg/mL h in the two-compartment model and the statistical moment in adult rats, respectively. There is no significant difference in AUC_(0-∞)_ of lenvatinib between adult and juvenile rats for the two-compartment model and the statistical moment. However, the MRT_(0-∞)_ was significantly 6.4 times increased in juvenile rats compared to those in adult rats.

**FIGURE 5:**
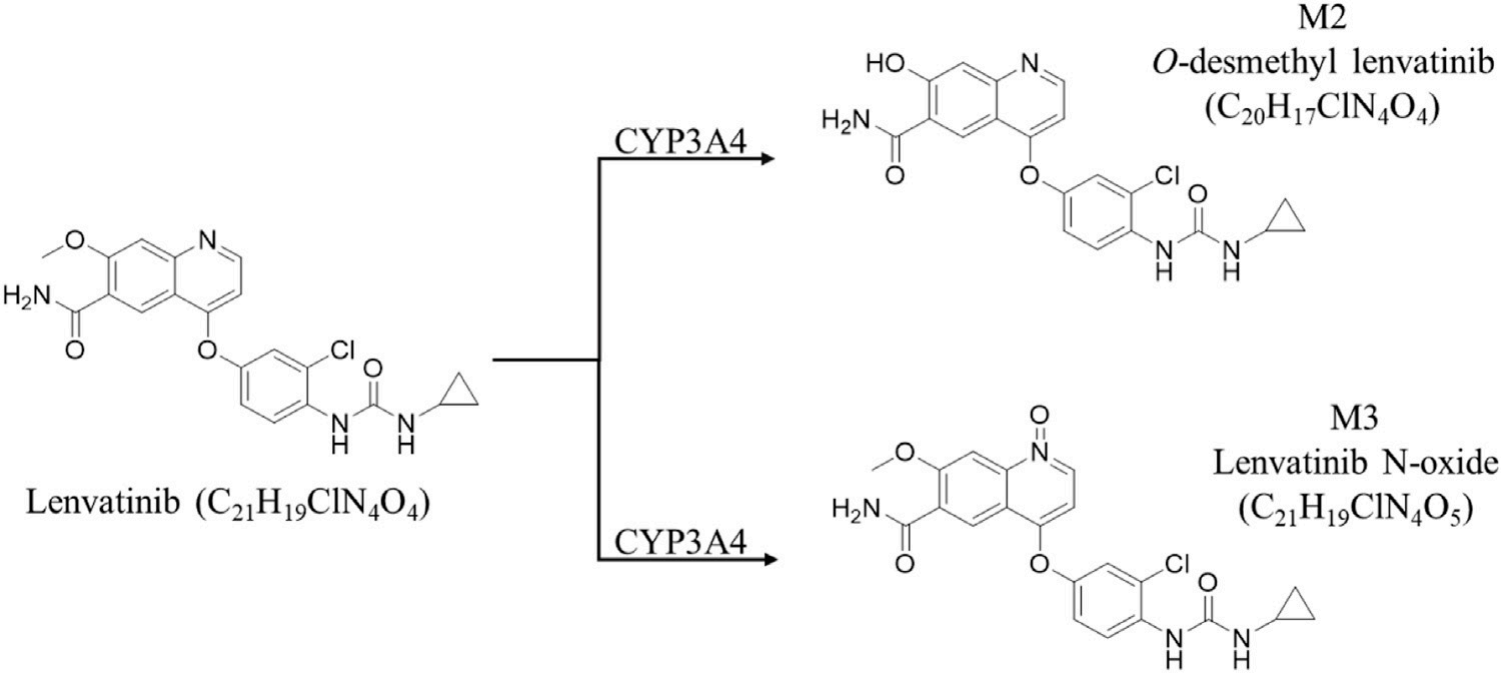
Lenvatinib is metabolised to two metabolites by CYP3A4 enzymes.

We speculate that the difference in MRT_(0-∞)_ due to age may further result to the difference in steady-state blood concentrations. Given that steady-state blood concentrations after multiple doses directly correlate with efficacy effects, we measured steady-state serum and liver lenvatinib levels in adult and juvenile rats by oral gavage for 5 consecutive days. The results showed there is no difference in steady-state serum lenvatinib levels between adult and juvenile rats. However, a significant decrease of steady-state liver lenvatinib levels and liver/serum content ratios were observed in juvenile rats compared with those observed in adult rats, suggesting that the metabolism or transporters of lenvatinib in the liver of adult and juvenile rats may be significantly different. Previous studies showed that cytochrome P450 is the main enzyme family involved in the drug metabolism of lenvatinib, of which mainly CYP3A4 is involved in human metabolism (Shumaker et al., 2014; Gupta et al., 2016; Vavrova et al., 2022). For rats, the corresponding metabolizing enzyme is CYP3A2, while lenvatinib is mainly produced in the presence of CYP3A4 enzyme as O-demethylated lenvatinib (M2-413.1) and N-decyclopropyl lenvatinib (M3-386.2) (Vavrova et al., 2022) (Figure 5). The SLC22A1 gene is strongly expressed mainly in the epithelial barrier and sinusoids of human and animal livers. SLC22A1 plays a key role in the efficacy of anticancer drug delivery (Arimany-Nardi et al., 2015). It has been reported (Herraez et al., 2013) that SCL22A1 is a transporter of sorafenib *in vivo*, suggesting that SLC22A1 may also be a transporter of lenvatinib, as a tyrosine kinase inhibitor like sorafenib.

Studies have shown that age is a key factor influencing P450s expression in rats, CYP3A1 and CYP3A2 were observed to be low in the fetal and neonatal stages, increased with age and exhibited rapid decrease with aging (Xu et al., 2019). For this reason, we further examined the mRNA expression levels of SLC22A1 and CYP3A2 in the liver of adult and juvenile rats. Our results showed that CYP3A2 levels were significantly higher in juvenile rats (4 weeks) than in adult (8 weeks) rats with a significant decrease in steady-state liver levels of lenvatinib. In addition, SLC22A1 was significantly higher in juvenile rats relative to adult rats, which have a greater ability of the liver to take up lenvatinib from the blood in juvenile rats, resulting in a higher liver/serum ratio in juvenile rats. However, significant decrease of steady-state liver lenvatinib levels and liver/serum content ratios were observed in juvenile rats compared with those observed in adult rats in this study. Thus, our result indicated that SLC22A1 may not be a transporter of lenvatinib in rats. The age difference in CYP3A2 expression is an important factor contributing to the significant differences in the pharmacokinetics and steady-state serum concentrations of lenvatinib in adult *versus* juvenile rats.

However, CYP3A showed appreciable interspecies differences (Tian et al., 2021). A recent study showed that the phase I metabolic characteristics of mulberrin in pigs and monkeys had significant differences compared with humans (Hu et al., 2022). The expression of SLC22 also depends on the species of the animal, which may seriously affect the pharmacokinetics of cationic drugs (Motohashi and Inui, 2013). *The* current *study was performed* using a *rat model*, and species differences between *rat* and human in the steady-state blood concentrations after lenvatinib treatment remains to be seen.

## 5 Conclusion

In this study, a UPLC-MS method for the determination of lenvatinib in rat serum and liver was developed and validated. The pharmacokinetic results of a single dose of lenvatinib showed a 6.4-fold decline in MRT_(0-∞)_, but no difference in AUC_(0-∞)_ in juvenile rats compared to those in adult rats. The mechanism of about 4-fold decreases in liver lenvatinib levels after multiple-dose administration was associated with age-dependent alteration of CYP3A2 expression. Our study may draw attention to and stimulate further study of the correct dosage in human patients to attain the desired clinical outcome of lenvatinib treatment.

## Data Availability

The raw data supporting the conclusions of this article will be made available by the authors, without undue reservation.
